# Supplementary material for: Bioprinted Schwann and Mesenchymal Stem Cell Co-Cultures for Enhanced Spatial Control of Neurite Outgrowth
Source: Gels. 2023 Feb 22;9(3):172. doi: 10.3390/gels9030172 (PMC10048219; doi:10.3390/gels9030172)
Supplement: Supplementary file 1 [file gels-09-00172-s001.zip › gels-2223099-supplementary.pdf]

Supplementary information

Bioprinted Schwann and Mesenchymal Stem Cell Co-Cultures for Enhanced Spatial Control of Neurite Outgrowth

Enateri V. Alakpa [1], Anton Bahrd [2], Krister Wiklund [2], Magnus Andersson [2], Lev N. Novikov [1], Christina Ljungberg [3,*] and Peyman Kelk [1,*]

[1] Department of Integrative Medical Biology, Umeå University, SE-901 87 Umeå, Sweden
[2] Department of Physics, Umeå University, SE-901 87 Umeå, Sweden
[3] Department of Surgical and Perioperative Science, Section of Hand and Plastic Surgery, Umeå University, SE-901 87 Umeå, Sweden
* Correspondence: christina.ljungberg@umu.se (C.L.); peyman.kelk@umu.se (P.K.)

Characterisation of mesenchymal stem cells (MSCs) and Schwann cells

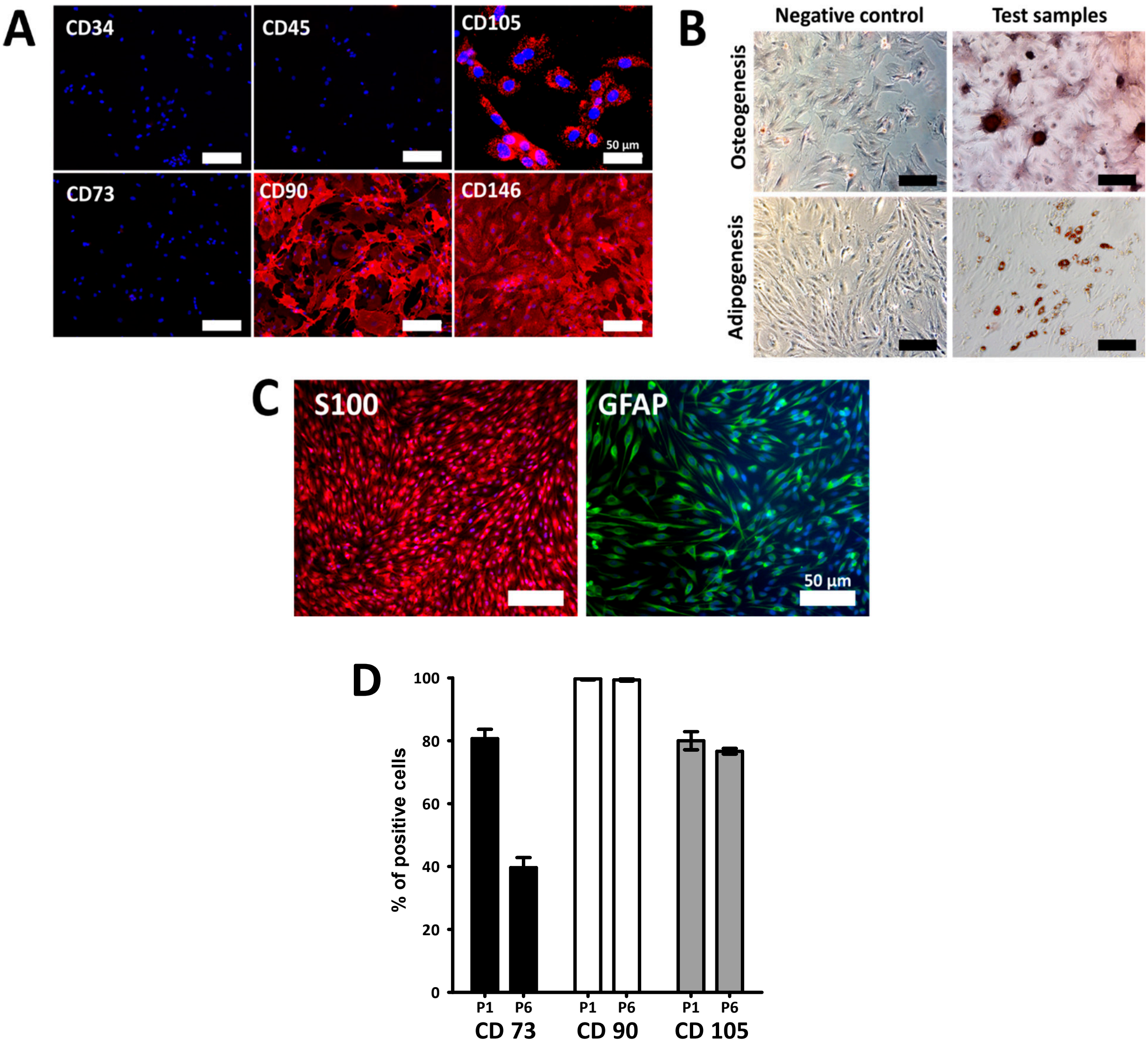

Supplementary Figure S1: Characterisation and differentiation of bone marrow derived adult mesenchymal stem cells (BMSCs) and Schwann cells derived from the sciatic nerve of the rat. Prior to use, BMSCs were expanded *in vitro* until passage 5 and immunofluorescently stained for the surface markers CD34, CD45, CD73, CD90, CD105 and CD146 (A). BMSCs were also induced to undergo osteogenesis and adipogenesis, cells were stained with alizarin red for bone nodules and oil red for fat droplets after three weeks in culture (B). Schwann cells (SCs) were also confirmed by immunofluorescently staining for S100 and glial fibrillary acidic protein (GFAP) (C). Scale bars are 100 µm except where stated otherwise. (D) Flow cytometry analysis of rat BMSC at passage 1 or passage 6 revealed that the expression of CD73 was decreased by time in culture, while the expression of CD90 and CD105 was unchanged over time. Error bars in D are standard deviations from the mean, n = 3.

Optimisation of the printing parameters

Cell damage depends on the magnitude and the exposure time of the shear stress [36] The exposure time is, in turn, a function of the fluid velocity implying that a non-slip surface boundary condition yields a slow-moving fluid (high exposure time) in the proximity of the wall. Our simulations of the bioink flow through a syringe with either a conical and cylindrical nozzle, indicate a maximum shear stress of 450 Pa and 200 Pa at the nozzle wall. Thus, using our simulation shear stress data that consider the exposure time, in combination with the cell damage data provided in [27], we can estimate the cell damage to be small in general. In the centre of the nozzles where we have the highest mass flow rate, we estimate cell damage to be less than 5%, whereas it is higher in the proximity of the wall, with a worst-case scenario of 30%. To have a good balance between printing resolution, flowrate and cell damage our simulations suggest using our 260 µm conical nozzle in the 3D-printer.

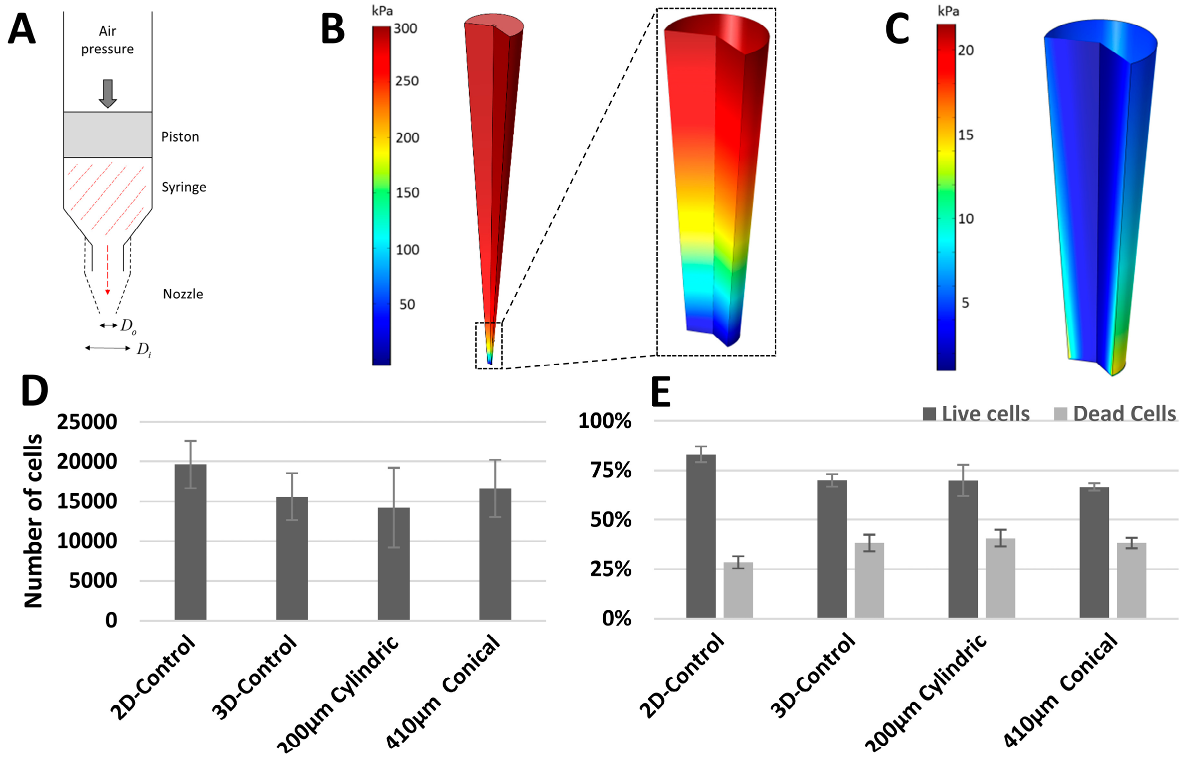

Supplementary Figure S2. *A*) a schematic of the bioprinter dispensing system showing pressure driven biomaterial (red) deposition through the interchangeable nozzle attachment (dashed lines). B) and C) shows heat maps of hydrostatic pressure and shear stress levels (Pa) for a 410 µm diameter conical nozzle (length 20mm) and a 200 µm cylindrical nozzle (length 12.7mm). Comparative cell analysis in non-printed (2D & 3D control) and printed biomaterial using cylindrical and nozzle design were ascertained. Total cell numbers acquired from each process and subsequent viability was ascertained using flow cytometry (D) and live/dead analysis (E). Error bars in D and E are standard deviations from the mean, n = 10.

Pseudoplastic properties of the biomaterial

The biomaterial used in the 3D-printer is described as a pseudoplastic non-Newtonian fluid by a power law model relating shear stress τ to the velocity shear rate as,

$$\tau = K\dot{\gamma}^n \quad \text{(Eq. S1)}$$

where K is the flow consistency index, $\dot{\gamma}$ is the shear rate and n is the power law index. The shear rate dependent effective viscosity of the biomaterial, μ_{eff}, can then be written as

$$\mu_{eff} = K\left(\dot{\gamma}\right)^{n-1}. \quad \text{(Eq. S2)}$$

Analytical models of biomaterial nozzle flow rates

Following [38] we validated the CFD simulations using analytical solutions for the volumetric flow rate Q for cylindrical and conical nozzle geometry.

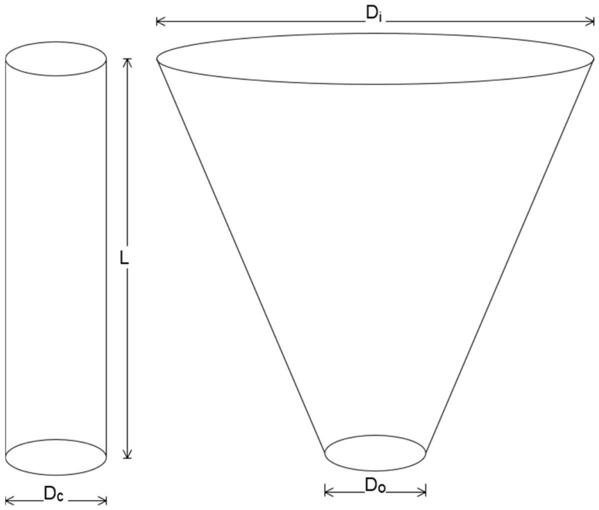

Supplementary Figure S3. A schematic of the two nozzles, cylindrical and conical, used in the CFD validation. The length of both geometries is denoted L, the outlet diameters for the cylinder and cone are D_c and D_o, respectively. The inlet diameter of the conical geometry is D_i and is assumed to be much larger than D_o.

In the case of incompressible fluid flow with a pseudoplastic behavior defined by Eq. (S1), the velocity profile V_c for a cylindrical geometry is given by

$$V_{cyl}(r) = \frac{n}{n+1}\left(\frac{-\Delta P D_c}{4KL_c}\right)^{1/n}\frac{D_c}{2}\left[1-\left(\frac{2r}{D_c}\right)^{(n+1)/n}\right], \quad \text{(Eq. S3)}$$

where r is the radial position in the cylindrical tube, ΔP is the pressure difference over the nozzle, D_c is the cylinder diameter and L is the cylinder length, as illustrated in Supplementary Figure S3.

In the case of a conical nozzle, assuming a power-law fluid defined by Eq. (S1), the flow rate Q_{con} can be expressed as

$$Q_{con} = \frac{\pi D_i^3 D_o^3}{32}\left[\frac{3n\Delta P \tan\theta_0}{2K(D_i^{3n} - D_o^{3n})}\right]^{1/n}, \qquad \text{(Eq. S4)}$$

where D_i is the inlet diameter, $D_o << D_i$ is the outlet diameter as illustrated in Supplementary Figure S3 and the half cone angle θ_0 is defined as,

$$\theta = \arctan\left(\frac{D_i - D_o}{2L}\right), \qquad \text{(Eq. S5)}$$

where L is the cone length.

Morphology of bioprinted MSCs and SCs in fibrin hydrogel

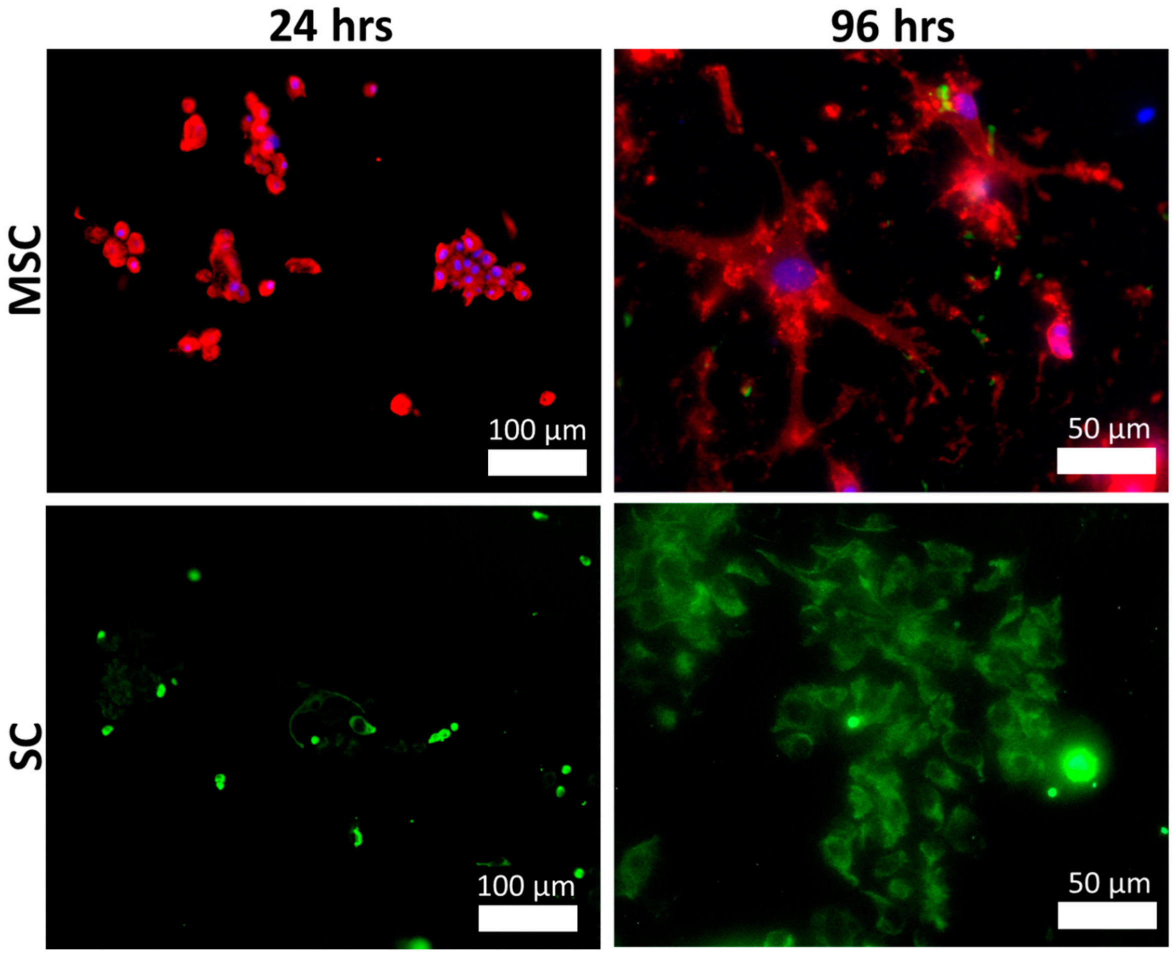

Supplementary Figure S4. Fluorescently stained bioprinted MSCs and SCs in fibrin 24 and 96 hours post printing. Cells were fluorescently stained for CD90 (MSCs) and GFAP (SCs) at 24 and 96 hours after printing onto PCL. Initial cell morphology at 24 hours were rounded. At 96 hrs, both MSCs and SCs adopted a more spread morphology. Schwann cells however remained somewhat rounded compared to the typical spindle like morphology that is observed on two dimensional planar surfaces like that in figure S1.

Organisation of neurite extensions from dorsal root ganglia (DRGs)

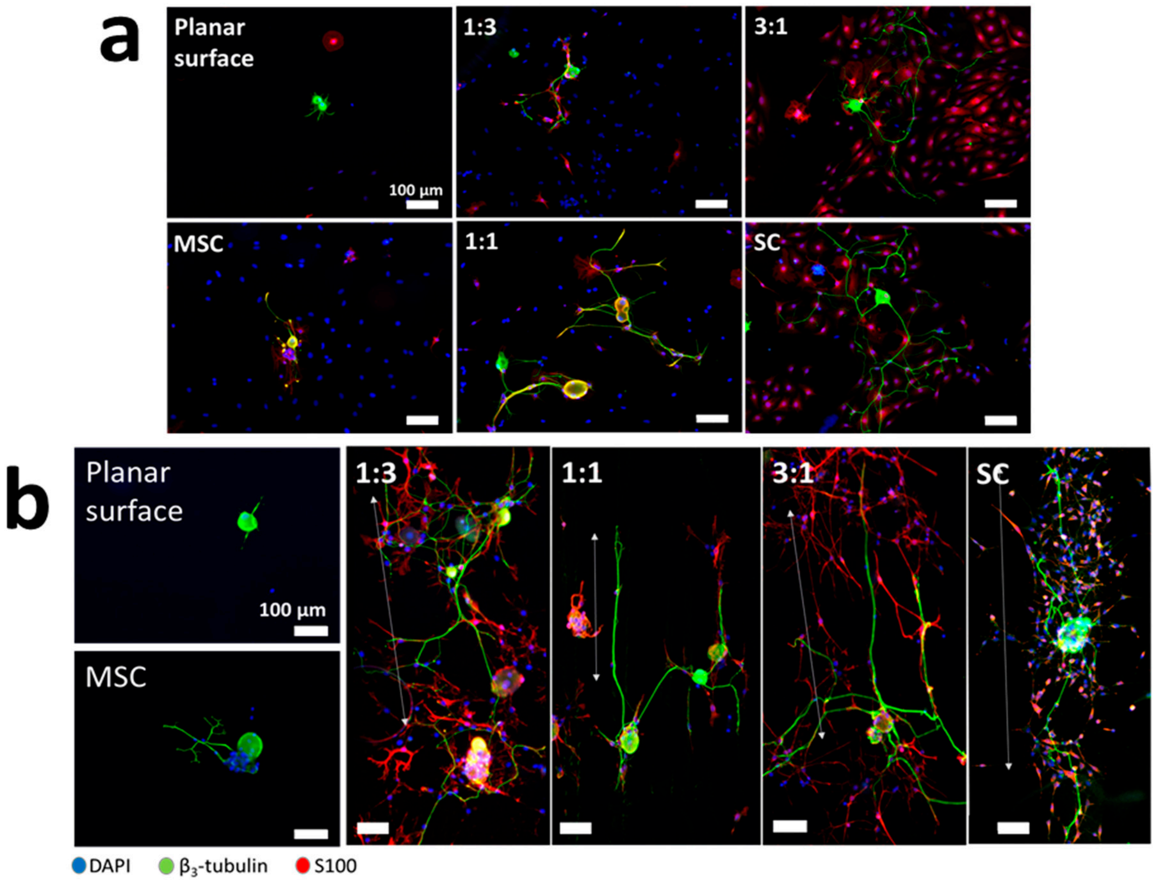

Supplementary Figure S5. Representative immunofluorescence images of neurites cultured on printed substrates. DRGs are cultured in the presence of MSC, SC or a co-culture of both in the ratios 3:1, 1:1 and 1:3. MSC/SC combinations were either seeded randomly onto a planar surface (2D substrate) or bioprinted to form fibrin tracks (3D substrate) on PCL substrate (A & B). Scale bars are 100 µm.

Neurite length and branching

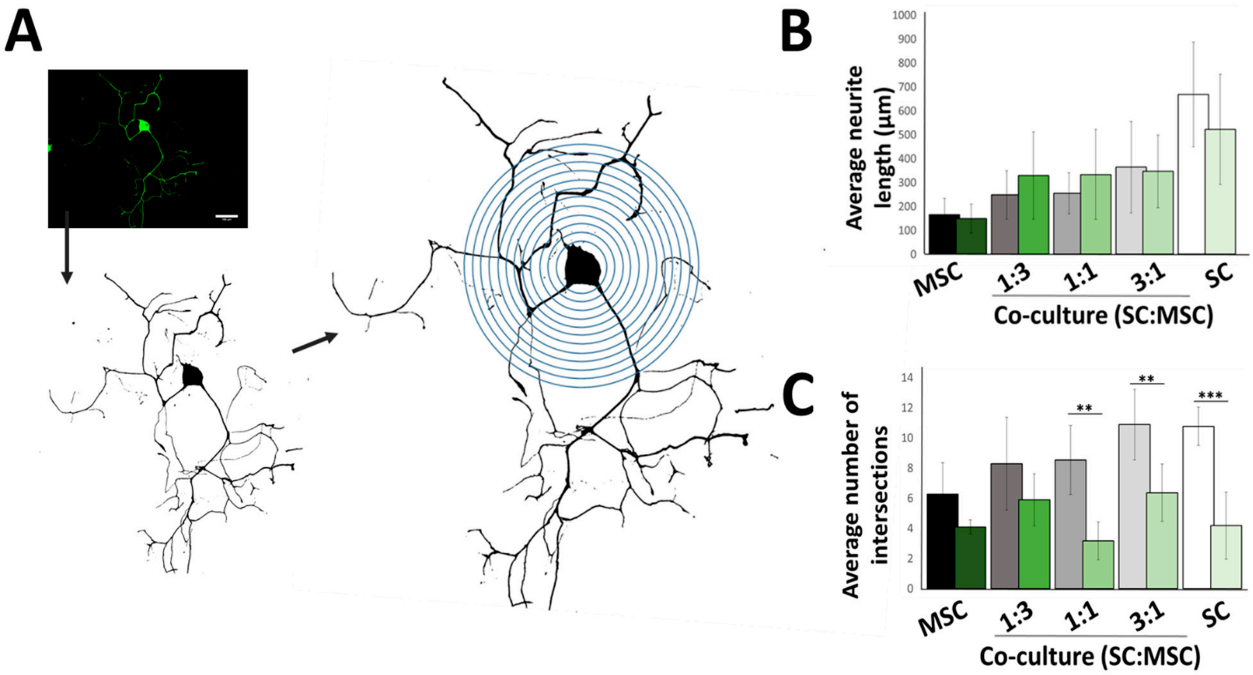

Supplementary Figure S6. Determination of overall neurite lengths and branching on both non-printed (2D) and bioprinted (3D) substrates. Representative thresholded image used for subsequent Sholl analysis as detailed in the methods to determine neurite branching is shown in (A). Prior to region segregation in S. Fig S6), average lengths of primary DRG neurites on flat (2D, shown in grayscale) and printed (3D, shown in 'greenscale') substrates are shown in (B). Average number of neurite intersection or sprouting points of DRG neurites on flat (2D) and printed (3D) substrates are shown in (C). Error bars are standard deviations from the mean; ** indicate significant difference between groups as determined by one-way ANOVA followed by Tukey post hoc test where $p < 0.01$, *** $p < 0.001$; $n \geq 5$. All scale bars are 100µm
